# Implementation of Strategies to Recognize and Control Hypertension in a Multispecialty Clinic, Montana, 2012–2013

**DOI:** 10.5888/pcd12.150116

**Published:** 2015-07-30

**Authors:** Julie Wall, Marilyn M. McLaury, Dorothy Gohdes, Carrie S. Oser, Crystelle C. Fogle, Steven D. Helgerson, Todd S. Harwell

**Affiliations:** Author Affiliations: Julie Wall, Benefis Health System, Great Falls, Montana; Dorothy Gohdes, Carrie S. Oser, Crystelle C. Fogle, Steven D. Helgerson, Todd S. Harwell, Montana Department of Public Health and Human Services, Helena, Montana.

## Abstract

Benefis Medical Group, in Great Falls, Montana, improved identification and treatment of hypertension through multifaceted interventions. The interventions included adopting policies for collection of vital signs, enhancing system-level reporting capability, tracking patients for the registry, and conducting patient outreach activities. From baseline to follow-up (December 2012 through September 2013), the percentage of patients with a documented blood pressure increased from 67% to 80%, the percentage diagnosed with hypertension increased from 16% to 36%, and the percentage with blood pressure control increased from 41% to 64%. Benefis Medical Group plans to sustain the successful evidence-based strategies that were adopted.

## Objective

High blood pressure is a major modifiable risk factor for heart disease, stroke, and kidney disease (1). One in 3 adult Americans has hypertension, but despite the availability of effective medication and lifestyle treatment, just over half of those patients with known high blood pressure have it controlled (2). Team-based care in combination with protocol-based treatment, patient registries, decision support systems, and patient outreach for self-care education and follow-up has been shown to improve hypertension control in a variety of settings (3). In December 2012, funds from Montana’s Community Transformation Grant were awarded to the Benefis Medical Group (BMG) in Great Falls, Montana, to begin a hypertension quality improvement project. This project aligned with BMG’s efforts to achieve Centers for Medicare and Medicaid Services Stage 1 Meaningful Use (4). These efforts would ultimately enable BMG to report Physician Quality Reporting System (PQRS) measure 236, Preventive Care and Screening: Screening for High Blood Pressure, an incentive to encourage health care systems to identify and control high blood pressure using their health information systems (5). This report describes the evidence-based strategies introduced during a 10-month period and the effect they had on improving identification and treatment of hypertension in a health care system in Montana.

## Methods

Benefis Health System is a nonprofit independent health care system based in Great Falls, Montana. The system, which uses NextGen (NextGen Healthcare Information Systems, LLC) electronic health record (EHR), includes a 516-bed hospital, an extended care and rehabilitation facility, and BMG, which consists of 130 employed physicians and advanced practice professionals and 3 primary care practices. BMG’s providers had an adult patient population of more than 13,000 during the study period (December 2012–September 2013). A multidisciplinary team worked on the project and included 15 health care providers from internal medicine, family practice and obstetrics and gynecology practices, along with personnel from administrative staff, information technology, and their quality improvement team. The team joined the American Medical Group Foundation’s Measure Up/Pressure Down blood pressure campaign and during 7 months participated in 10 online conferences (6). As part of this campaign, BMG was given access to a provider toolkit that included patient educational material. The best practice information for providers was used to guide their activities (3).

BMG implemented selected evidence-based strategies to improve blood pressure identification and control (Table). All vital signs, including blood pressure measures, had to be collected at each visit, and the data entry fields for the blood pressure measure were mandatory. BMG also began using automated vital sign monitors to download the blood pressure measurements directly into the EHR. The information technology staff developed provider and system-level reporting capability for the PQRS measure 317, Preventive Care and Screening: Screening for High Blood Pressure. In addition, BMG piloted the development of a hypertension registry to track and document outreach to patients and compliance with follow-up appointments. Plans were also initiated for the development of a patient portal that allows patients access to their information.

**Table:** Strategies Used to Improve Quality of Care for Treatment of Hypertension at a Multispecialty Clinic, Montana, 2012–2013

Strategies Used	
Electronic health record (EHR) support	• Included blood pressure (BP) screening at every visit as a mandatory field in the electronic health record (EHR).
	• Established baseline and post-project rates of patients who had their BP recorded at the time of the office visit, patients who had a diagnosis of hypertension, and patients who had a diagnosis of hypertension and whose hypertension was uncontrolled (>140/90 mm Hg).
	• Developed a registry of patients with controlled and uncontrolled diagnosed hypertension.
Hypertension team-based protocol	Introduced automated BP measurement devices for routine clinic use.
	• Distributed treatment protocols from provider toolkit.
Feedback at provider and system level	• Developed and disseminated monthly provider and system-level feedback reports using information from the hypertension registry about the prevalence of BP screening among all patients, the prevalence of patients with a diagnosis of hypertension, and the prevalence of patients with uncontrolled hypertension.
Patient outreach	• Conducted community outreach and education promoting wellness and preventive screenings.
Training	• Provided training to direct care staff in taking accurate BP measurement.
	• Provided clinicians access to current BP guidelines and protocols.
	• Participated in 10 online trainings provided through the Measure Up/Pressure Down Campaign.

## Results

During the 10-month period, the percentage of patients with a documented blood pressure increased from 67% at baseline to 80% after the project, and the percentage of adult patients diagnosed with hypertension increased from 16% to 36%, resulting in more than 4,000 adult patients with a hypertension diagnosis. In addition, blood pressure control improved by 23 percentage points, from 41% at baseline to 64% after the project.

## Discussion

BMG adopted evidence-based strategies for identifying and controlling hypertension for their population using best practices supported by its EHR. The providers became aware of blood pressure control as an issue to be addressed at each visit. Patients with elevated blood pressures were formally diagnosed and tracked. The efforts proved to be not only sustainable but were subsequently expanded to the larger patient population that included 2 specialties. The Community Transformation Grant enabled BMG to develop the IT infrastructure and the clinical hypertension control strategies that were used to report PQRS Measure 317, Screening for High Blood Pressure, as part of attesting to the Meaningful Use incentives program. BMG was able to expand the strategies to additional practices within BMG in subsequent years so they could begin reporting PQRS 236, Controlling High Blood Pressure, for the whole group.

There are important limitations to the findings. Patients lacking blood pressure readings during the measurement period could have been classified as having uncontrolled hypertension, thus affecting the overall improvement.

In summary, the seed grant provided the impetus for a small group practice to review, adapt, and successfully adopt the necessary activities in a stepwise fashion to improve the recognition and control of hypertension in their patient population, an important public health goal. BMG plans to sustain the successful evidence-based strategies that were adopted.
